# Analysis of Development Trends of the Research Hotspots of Vitamin D in Children

**DOI:** 10.3389/fped.2022.899844

**Published:** 2022-05-06

**Authors:** Xuemei Luo, Feifeng Wu, Cheng Wang, Chuan Wen

**Affiliations:** Department of Pediatrics, The Second Xiangya Hospital, Central South University, Changsha, China

**Keywords:** vitamin D, children, visualization study, MeSH, health

## Abstract

**Objective:**

Using multivariate statistics and social network analysis techniques, we present a realistic and intuitive visualization of the research hotspots and development trends of vitamin D in children.

**Methods:**

The Medical Subject Headings (MeSH) term “vitamin D” was used to search all the publications (the study subjects were 0–18 years old) included in PubMed by time period. The subject terms for each development stage were extracted, the high-frequency subject terms were extracted using the Bibliographic Items Co-occurrence Matrix Builder (BICOMB), and a core subject term co-occurrence matrix was established. The Netdraw function of Ucinet 6.0 software was used to complete the social network drawing of the core subject term co-occurrence matrix to form a co-word network diagram composed of core subject terms.

**Results:**

Prior to 1979, there were 890 papers with 1,899 core subject terms; from 2010 to 2020, there were 3,773 papers with 12,682 core subject terms. Before 1979, the research direction of vitamin D in children focused on vitamin D in the classical regulation of calcium and phosphorus metabolism. From 1980 to 1989, studies focused on vitamin D metabolites and therapeutic drugs such as “calcitriol” and “calcifediol.” From 1990 to 1999, studies focused on “calcitriol” and its association with “psoriasis,” “chronic renal failure,” and “dermatological drugs.” From 2000 to 2009, studies focused on “vitamin D” and “vitamin D deficiency.” From 2010 to 2020, studies focused on “vitamin D_3_” and its association with “vitamins,” “bone mineral density protectants,” “asthma,” “obesity,” “pregnancy complications” and “fetal blood.”

**Conclusion:**

Since 2010, the research direction of vitamin D in children has been growing rapidly, and the overall development trend is good. Studies extend from the study of the skeletal effect of vitamin D to the study of its extraskeletal effect and the investigation of mechanisms of its association with related diseases.

## Introduction

Vitamin D deficiency is the most common nutrient deficiency. Globally, ~30% of children and 60% of adults have vitamin D deficiency (1, 2). The physiological effects of vitamin D (1, 3–9), in particular, the extraskeletal effect, has attracted increasing attention from researchers (10–27). As an important method in bibliometrics, multivariate statistics and social network analysis techniques can help researchers better grasp the evolutionary trend of a field of interest and think in-depth about the research scope. To further understand the research situation in the field of vitamin D in children, this study used multivariate statistics and social network analysis techniques to analyze the published studies and visualize the historical research, evolutionary paths and direction trends in a realistic and intuitive way.

## Materials and Methods

### Literature Database Search

All publications included in the PubMed database retrieved using relevant subject terms were analyzed.

### Principles for Establishing Core Subject Terms

Terms with a word frequency greater than 0.5% were set as the core subject terms and used in the subsequent analysis.

### Search Strategy

#### Subject Terms

Vitamin D [MeSH Major Topic]

#### Staged Retrieval

The retrieval periods were classified according to natural chronology. The first stage was before December 31, 1979 (given the limited number of documents included in the PubMed database in the early years, the period before 1979/12/31 is classified as the same stage); the retrieval period for the second stage was [“1980/01/01” (Date-Publication): “1989/12/31” (Date-Publication)]; the retrieval period for the third stage was [“1990/01/01” (Date-Publication): “1999/12/31” (Date-Publication)]; the retrieval period for the fourth stage was [“2000/01/01” (Date-Publication): “2009/12/31” (Date-Publication)]; and the retrieval period for the fifth stage was [“2010/01/01” (Date-Publication): “2020/12/31” (Date-Publication)].

#### Study Subjects

The following terms were used for retrieving studies pertaining to the age groups of interest: “child: birth−18 years” [MeSH Terms] OR “newborn: birth−1 month” [MeSH Terms] OR “infant: birth−23 months” [MeSH Terms] OR “infant: 1–23 months” [MeSH Terms] OR “preschool child: 2–5 years” [MeSH Terms] OR “child: 6–12 years” [MeSH Terms] OR “adolescent: 13–18 years” [MeSH Terms].

### Multivariate Statistical and Social Network Analysis Methods

The searched publications were imported from the PubMed database into the Bibliographic Items Co-occurrence Matrix Builder (BICOMB) (28). All the subject terms for the five development stages were extracted; BICOMB was used to extract the high-frequency subject terms, establish the core subject terms, and finally establish the core subject term co-occurrence matrix. The Netdraw function of Ucinet 6.0 software was used to complete the social network drawing of the core subject term co-occurrence matrix to form a co-word network diagram composed of core subject terms. In the social network diagram of the core subject terms, the closer is the node to the center, the more the topic is in a core position in the social network; the larger is the node, the higher is the word frequency; and the thicker is the line between the core subject terms, the higher is the correlation between the two core subject terms.

## Results

### General Information of the Retrieved Literatures

**Figure 1:**
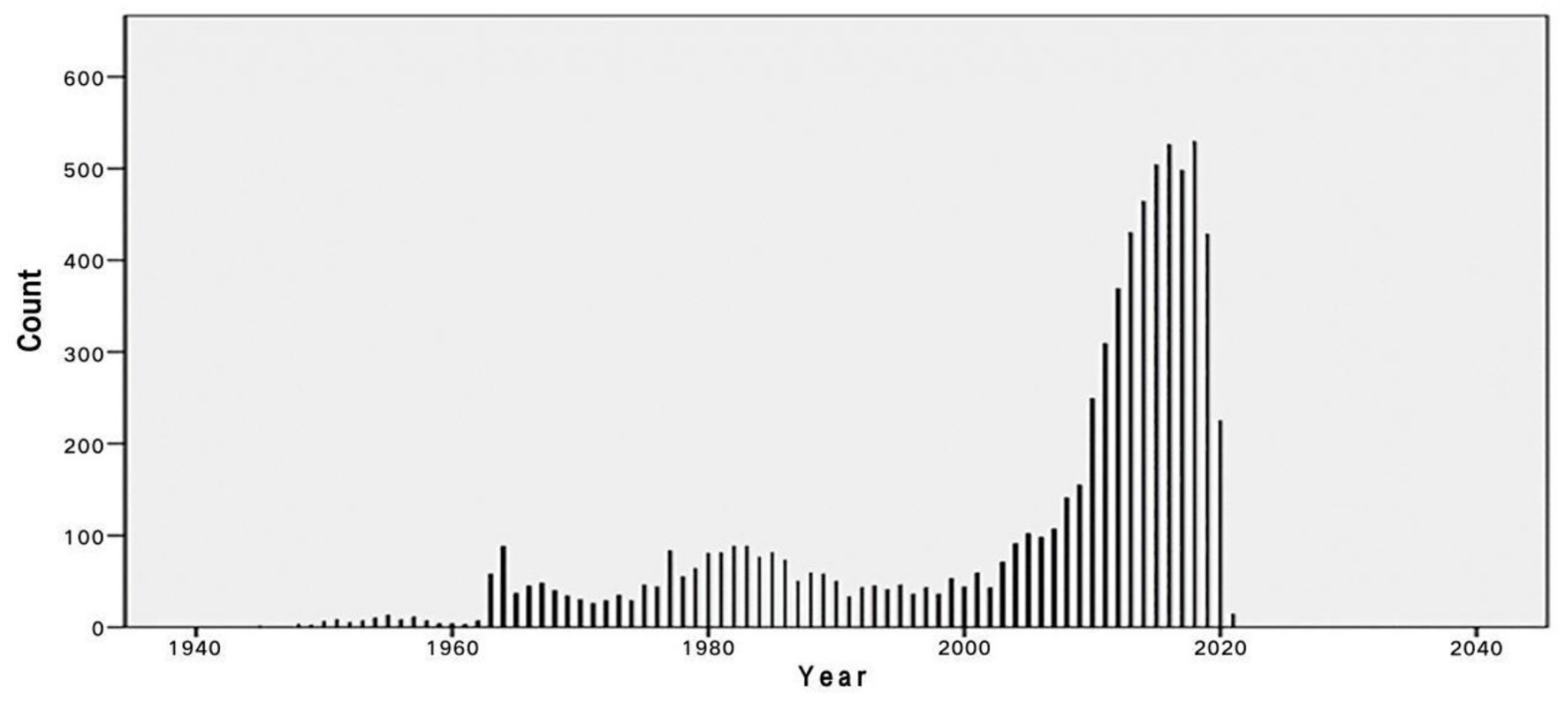
Number of publications on “vitamin D (children)” in the PubMed database as of December 2020.

We searched the PubMed database and selected subject terms with a word frequency greater than 0.5% as the core subject terms. For the time period before 1979, 890 publications were retrieved, with 1,899 main subject terms; the lowest core subject term frequency was 10, and 30 subject terms were analyzed. A total of 734 publications from 1980 to 1989 were retrieved, with 2,143 main subject terms; the lowest core topic word frequency was 11, and there were 33 subject terms included in the analysis. From 1990 to 1999, 436 publications were retrieved, with 1,400 main subject terms; the lowest core topic word frequency was 7, and 31 subject terms were included in the analysis. For the time period from 2000 to 2009, 859 publications were retrieved, with 2,894 main subject terms; the lowest core subject term frequency was 16, and 26 subject terms were included in the analysis. For the time period from 2010 to 2020, 3,773 publications were selected, with 12,682 main subject terms; the lowest core subject term frequency was 73, and 19 subject terms were included in the analysis (Figure 1; Table 1).

**Table 1:** Literature analysis of “vitamin D (children)” in PubMed as of December 2020.

**Stage**	**No. of retrieved publications**	**No. of main subject terms**	**Social network analysis of core subject terms**		
			**Core**	**Second layer**	**Outer layer**
~1979	890	1,899	Vitamin D	Calciferol	Vitamin D deficiency
			Calcium	Cholecalciferol	Vitamin A
			Hydroxycholecalciferol	Dihydrotachysterol	Phosphate
				Osteomalacia	Familial hypophosphatemia
				Rickets	Alkaline phosphatase
				Pseudohypoparathyroidism	Kidney
					Chronic renal failure
					Bone diseases
					Prednisone
					Anticonvulsants
					Infant/premature infant disease
1980–1989	734	2,143	Vitamin D	Dihydroxycholecalciferol	Osteoporosis
				Calciferol Hydroxycholecalciferol	Osteomalacia Anticonvulsants
					Fetal blood
				Calcitriol	
				Calcifediol	
				Rickets	
1990–1999	436	1,400	Calcitriol	Psoriasis	Vitamin A
				Dermatological drugs	Osteoblasts
				Chronic renal failure	Osteocalcin
					Nephrocalcinosis
					Uremia
					Calcium and diet
					Growth disorders
2000–2009	859	2,894	Vitamin D	Vitamin D deficiency	Cystic fibrosis
					Vitiligo
					Type 1 diabetes
					Vitamin D receptor
					Betamethasone
					Anti-inflammatory drugs
2010–2020	3,773	12,682	Vitamin D_3_	Calcidiol	Obesity
				Vitamins	Pregnancy complications
				Bone density protectant	Fetal blood
				Asthma	
				Calcitriol receptor	
				Calcium and diet	

### Social Network Analysis of the Core Subject Terms

**Figure 2:**
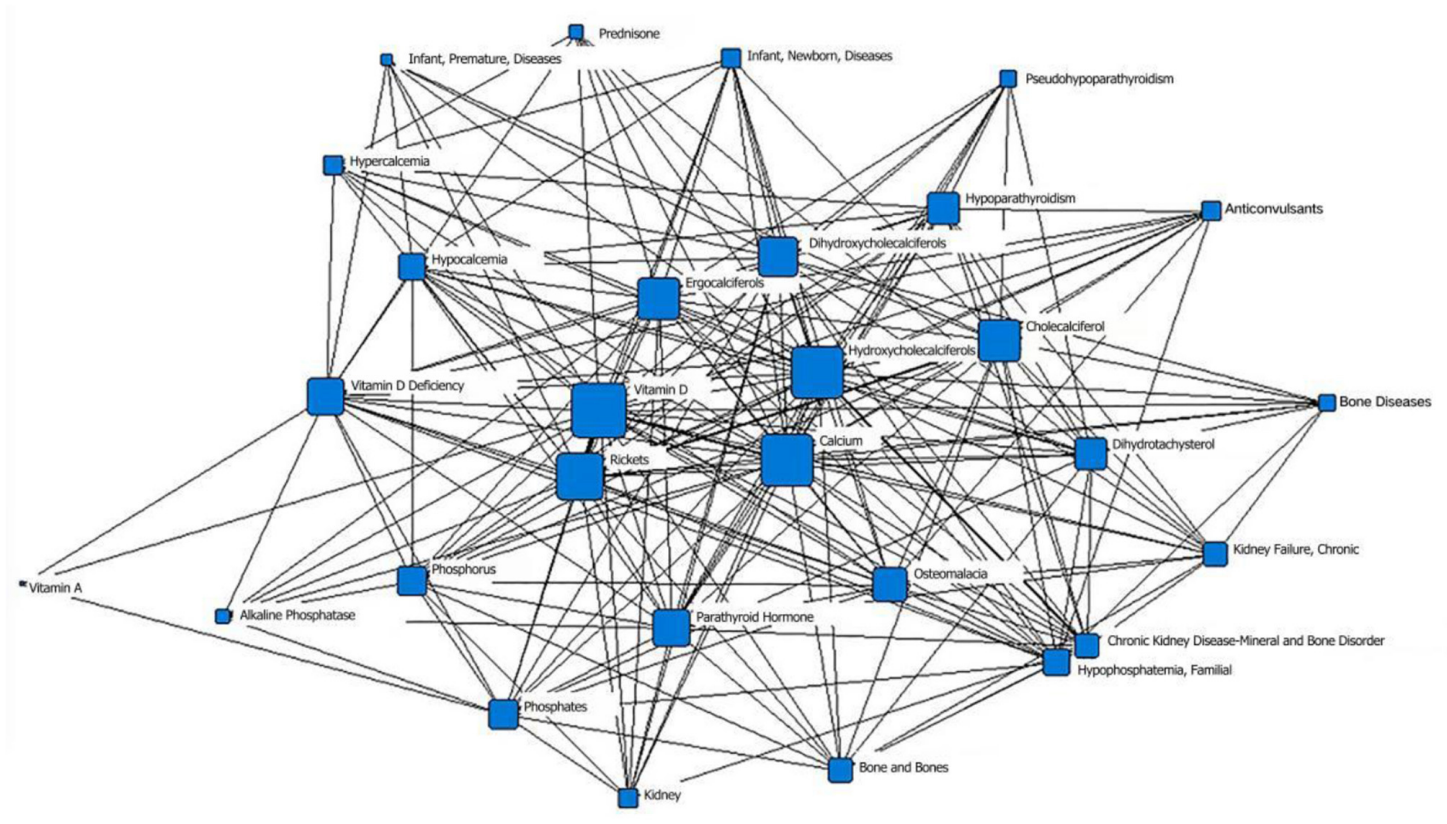
Social network of core subject terms of “vitamin D (children)” in the PubMed database before December 1979.

In the first stage (before 1979), the core positions were “vitamin D,” “calcium,” and “hydroxycholecalciferol.” Their node areas were all large and closely linked to the surrounding subject terms. The second layer of subject terms included “calciferol,” “cholecalciferol,” “dihydrotachysterol,” diseases caused by calcium and vitamin D deficiency (such as “osteomalacia” and “rickets”), and hypocalcemia-related diseases (such as “pseudohypoparathyroidism”), etc. “vitamin D deficiency,” “vitamin A,” “phosphate,” “familial hypophosphatemia,” “alkaline phosphatase,” “kidney,” “chronic renal failure,” “bone disease,” and “prednisone,” “anticonvulsant drugs,” “infants, premature infants, and diseases” were all located in the outer layer of the network (Figure 2; Table 1).

**Figure 3:**
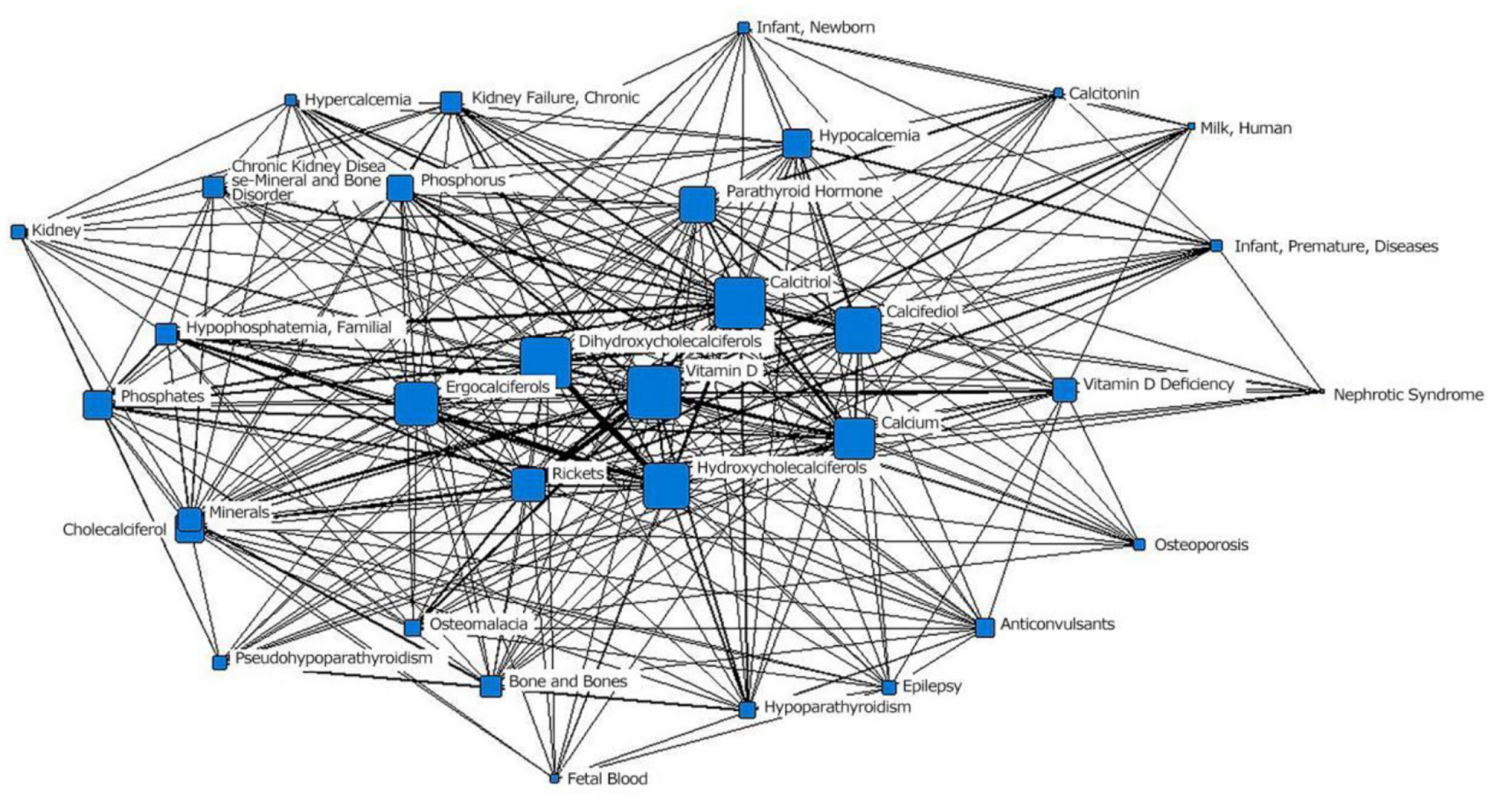
The social network of the core subject terms of “vitamin D (children)” in the PubMed database from 1980 to 1989.

In the second stage (1980–1989), “vitamin D” was located at the core position, with the largest node area, of the social network of the core subject terms and was closely related to the surrounding subject terms. The second layer mainly included vitamin D metabolites such as “dihydroxycholecalciferol,” “calciferol,” and “hydroxycholecalciferol” and therapeutic drugs such as “calcitriol” and “calcifediol”; the node area of “rickets” decreased. “Osteoporosis,” “osteomalacia” and other related diseases and “anticonvulsant drugs” that may affect the level of vitamin D in the body were in the outermost layer. The term “fetal blood” appeared for the first time in the network diagram (Figure 3; Table 1).

**Figure 4:**
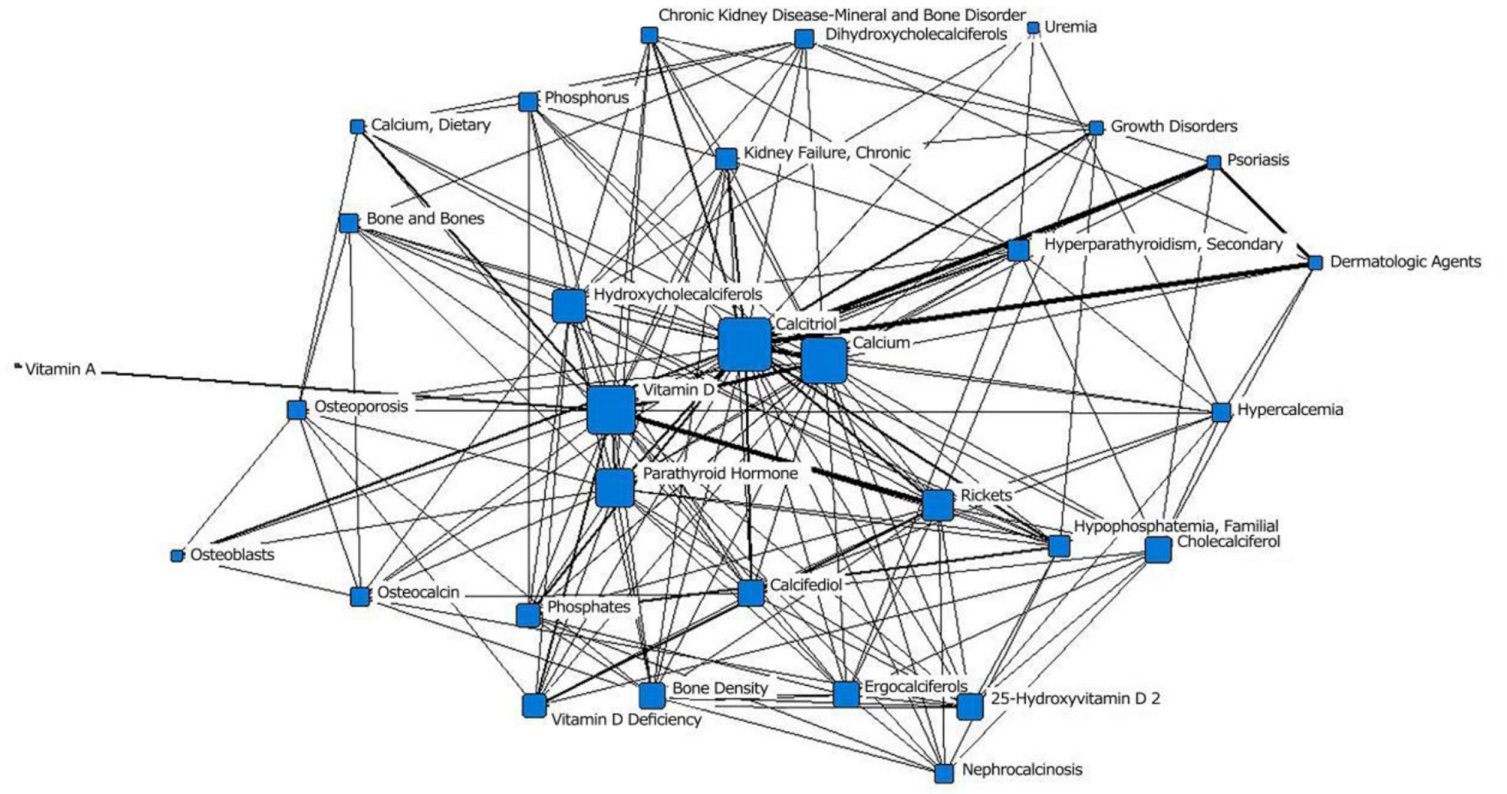
Social network of the core subject terms of “vitamin D (children)” in the PubMed database between 1990 and 1999.

In the third stage (1990–1999), “calcitriol” was located at the core of the network and had the largest node area. It was closely related to surrounding subject terms. Among those terms, the connections with the two subject terms “psoriasis” and “dermatological drugs” were the thickest, indicating the closest relationships. The term “chronic renal failure” moved from the outer layer to the middle layer. “Vitamin A” was still in the outer layer of the network. “osteoblasts,” “osteocalcin,” “nephrocalcinosis,” “uremia,” “calcium and diet,” and “growth disorders” first appeared in the network (Figure 4; Table 1).

**Figure 5:**
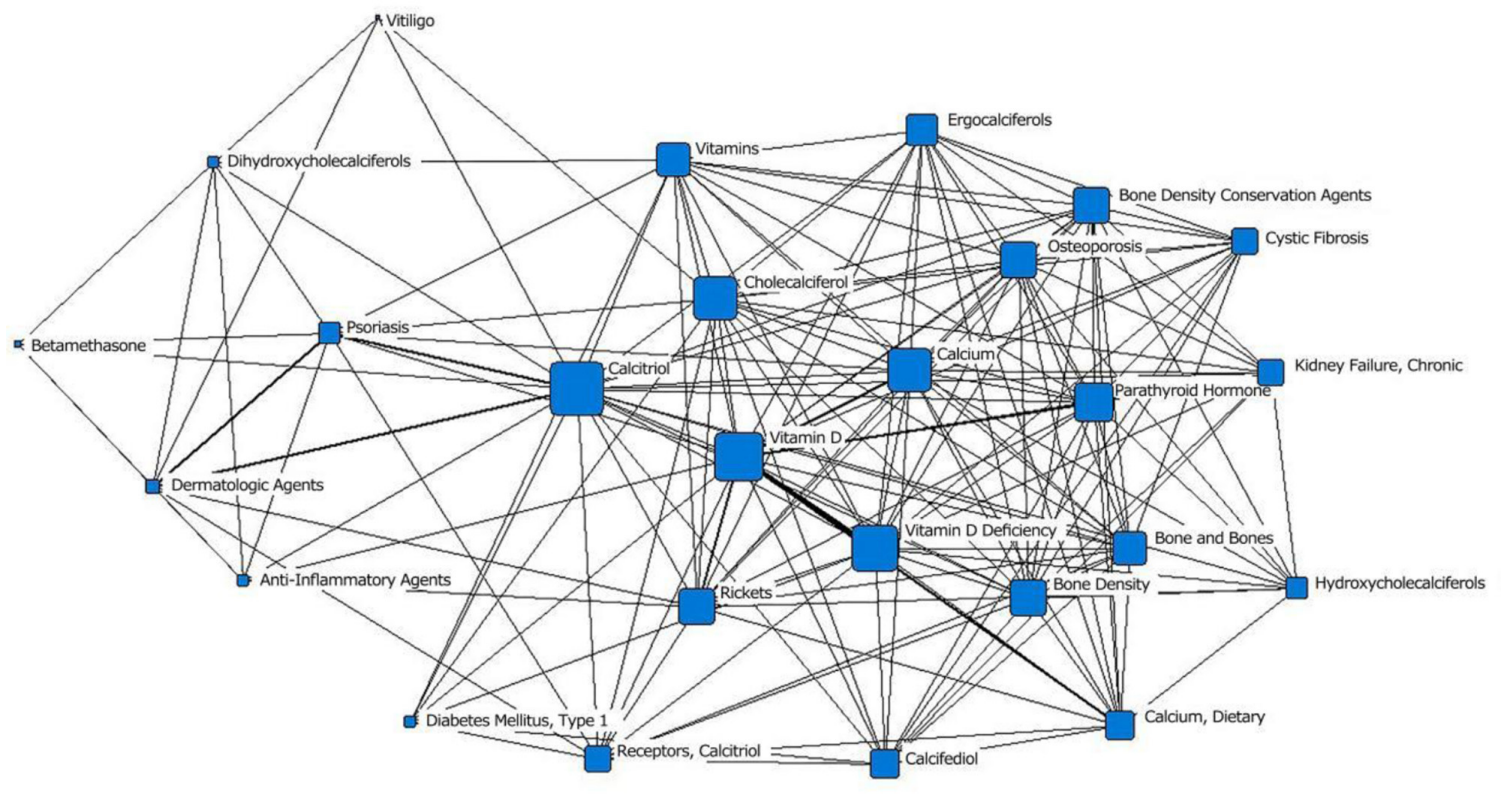
The social network of the core subject terms of “vitamin D (children)” in the PubMed database from 2000 to 2009.

In the fourth stage (2000–2009), “vitamin D” was located at the core position and had the largest node area. It was closely related to surrounding subject terms. “vitamin D deficiency” moved to the second layer from the outer layer, and the number of research studies increased. “cystic fibrosis,” “vitiligo,” “type 1 diabetes,” “vitamin D receptor,” “betamethasone,” and “anti-inflammatory drugs” first appeared in the outermost layer (Figure 5; Table 1).

**Figure 6:**
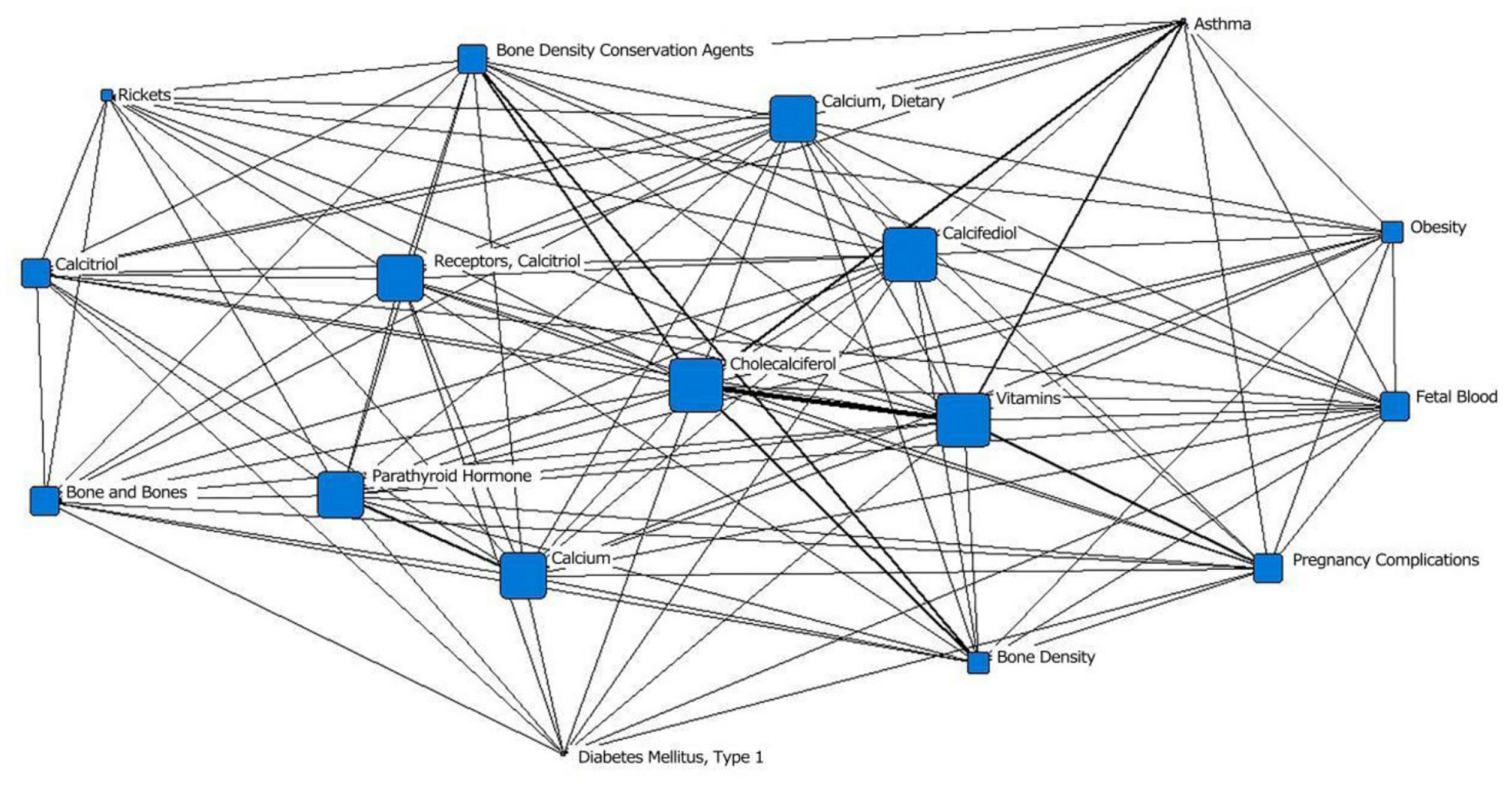
Social network of the core subject terms of “vitamin D (children)” in the PubMed database from 2010 to 2020.

In the fifth stage (2010–2020), “vitamin D_3_” was located at the core of the network, and the area of its node was slightly smaller than that of “calcidiol.” It was closely related to surrounding subject terms, with the closest relationships with “vitamin,” “bone density protectant” and “asthma.” “Calcitriol receptor” was in the middle layer; in the previous stage, it was in the outermost layer. The area of the “calcium and diet” node was significantly larger than that in the previous stage. “obesity” and “pregnancy complications” first appeared in the outermost layer of the network; “fetal blood,” which appeared in the second stage, appeared again (Figure 6; Table 1).

## Discussion

In this study, the evolving trends of research on vitamin D in children was analyzed using a bibliometric analysis method. The results showed that the number of studies in this research field has increased rapidly since 2010. The number of publications (3,778) retrieved for the 2010 to 2020 period was 4.2 times the number of publications (890) retrieved for the period before 1979, and the overall development trend is good. The social network analysis of the core subject terms showed that the research hotspots of vitamin D in children gradually extended from a focus on the effects of vitamin D on classic calcium and phosphorus metabolism to the exploration of its multisystem effects and the mechanisms of its association with related diseases (such as diabetes, kidney disease, asthma, and obesity). These studies reveal the extensive physiological functions and research prospects of vitamin D.

The first stage (before 1979) of research focused on the classic bone effects of vitamin D. “osteomalacia” and “rickets,” which are caused by vitamin D deficiency, also received attention. As early as 1634, it was reported that sunlight exposure and cod liver oil supplementation can lead to recovery from rickets (3). “Infant, premature infant, and disease” was at the outermost layer of the network, indicating that vitamin D deficiency in infants, especially preterm infants, received attention from researchers. Infants and adolescents are prone to vitamin D deficiency due to rapid growth. The low vitamin D content in breast milk leads to a high incidence of rickets in breastfed infants. Compared with full-term infants, preterm infants are more likely to develop nutritional vitamin D deficiency rickets (3, 29), which is related to low vitamin D levels during late pregnancy and insufficient sunlight exposure due to hospitalization after birth.

In the second stage (1980–1989), “fetal blood” appeared in the network transiently and reappeared in the fifth stage (2010–2020). The maternal vitamin D concentration during pregnancy is correlated with the risk of neonatal vitamin D deficiency. By measuring the serum 25(OH)D level of pregnant women at early (<16 weeks, T1), mid- (24–28 weeks, T2), and late (32–34 weeks, T3) pregnancy as well as the corresponding cord blood in the newborns, Wang et. al reported that neonatal 25(OH)D level in cord blood was positively correlated with maternal serum 25(OH)D levels at each trimester, and the strongest correlation was found at time point T3 (30). In one study, prenatal serum 25(OH)D ≤ 27.55 ng/ml was used as the cutoff value to predict neonatal vitamin D deficiency, with a sensitivity of 97.2% and a specificity of 80.3%. These results suggest that neonatal vitamin D deficiency can be predicted by the prenatal serum 25(OH)D concentration (31). The correlation was also confirmed in another study, which also found that maternal vitamin D deficiency was associated with low vitamin D level in cord blood (32). Meanwhile, it is currently believed that low neonatal 25(OH)D concentration (<25 nmol/L) was associated with an increased risk of preterm delivery, neonatal respiratory distress syndrome, and hospitalization during the first year of life because of acute respiratory infection or gastroenteritis (33). In addition, maternal 25(OH)D had an inverted U-shaped relationship with cord blood insulin and c-peptide. This suggested that vitamin D may play a role in regulating fetal insulin secretion and impacting glucose regulation and growth (34). The re-emergence of this study indicates that the intergenerational connection of vitamin D deficiency has regained attention. Although the significant effect of vitamin D nutritional status on fetal bone development is widely recognized, the literature on the recommended dose of vitamin D supplementation during pregnancy is conservative because the ideal threshold for vitamin D during pregnancy is unclear (35).

Research on the extraskeletal effects of vitamin D gradually became popular. In the third stage (1990–1999), there was a significant increase in the number of studies on “calcitriol” associated with “psoriasis,” “chronic renal failure,” and “dermatological drugs,” and “growth disorders” entered the network. In the fourth stage (2000–2009), studies on the association of “vitamin D” with “cystic fibrosis,” “vitiligo,” and “type 1 diabetes” first appeared in the outermost layer of the network. In the fifth stage (2010–2020), the association between “vitamin D_3_” and “asthma” received more attention, the number of studies on “calcitriol receptor” increased, and “obesity” and “pregnancy complications” appeared in the outermost layer of the network. Vitamin D has been shown to be associated with acute/chronic infectious diseases, cardiovascular diseases, kidney diseases, metabolic syndrome (obesity and type 2 diabetes), autoimmune diseases (asthma, systemic lupus erythematosus, multiple sclerosis, and Hashimoto's thyroiditis), tumors (skin cancer, colon cancer, kidney cancer, brain tumors, etc.), neurodegenerative diseases, brain development disorders, mental disorders (schizophrenia, autism, Alzheimer's disease, etc.) and other diseases (3, 10–27, 36–43). Obese people participate in fewer activities outdoors, have insufficient exposure to sunlight, have different dietary preferences, have impaired adipose tissue hydroxylation, and accumulate 25(OH)D in adipose tissue, which may be associated with a high incidence of vitamin D deficiency in this population. Patients with severe liver and kidney diseases are prone to vitamin D deficiency due to impaired vitamin D hydroxylation and 25(OH)D production disorders (22). Chronic liver and kidney diseases and the use of special drugs also have substantial impacts on the levels of vitamin D and its active products in the body.

The classical bone effect of vitamin D is still being studied. As the earliest recognized vitamin D deficiency disease, rickets is still reported in the fifth stage (2010–2020), showing a trend of “rewarming.” The trend of research hotspots was from the identification of nutritional rickets (mid-17th century), to the exploration of pathogenesis (in the 1920s, researchers gradually recognized that lack of light and insufficient dietary intake of vitamin D could lead to nutritional rickets), to the study of the effects of interventions with fortification (decrease in typical cases), to the discovery of differences in the pathogenesis and probability of nutritional rickets in different regions and ethnic groups (vitamin D deficiency is the main cause in some Asian and Middle Eastern countries, while calcium deficiency is more common in Africa and other Asian countries) (44). Vitamin D and calcium deficiency has become a global public health problem. A global consensus on nutritional rickets was developed by a consortium of 33 experts in pediatrics, pediatric endocrinology, epidemiology, nutrition, public health and health economics, representing 11 international scientific organizations. The consensus provides an evidence-based grading of the concept, diagnosis, treatment, and prevention of nutritional rickets in children, which provide guidance for the work of health care professionals and relevant policy makers in this field (45).

The severe acute respiratory syndrome coronavirus 2 that causes coronavirus disease 2019 (COVID-2019) outbreak and has spread worldwide rapidly since 2019, placing a significant burden on global healthcare systems. COVID-19 did not appear in the fifth stage due to the paucity of relevant literature. Given the serious impact of COVID-19 on human health, studies of the association between vitamin D in children and COVID-19 was discussed here. Rosa et. al reported that the implementation of home isolation during the COVID-19 pandemic resulted in the reduction of outdoor light exposure, which may elevate the risk of vitamin D deficiency especially in infants (46). A decreasing trend of serum 25(OH)D level was observed in the subsequent months post-outbreak at a monthly decline rate of −6.32 nmol/L. Thus guidelines was required to ensure sufficient vitamin D level in pregnant women and infants during the COVID-19 pandemic. Alpcan et al. (47) found that patients with COVID-19 had significantly higher rates of vitamin D deficiency than controls. In a retrospective analysis of 103 children with COVID-19, inflammatory markers (C-reactive protein, procalcitonin, fibrinogen, D-dimer) were significantly elevated in children with moderate to severe clinical signs, while serum 25(OH)D level was significantly lower. Vitamin D deficiency was present in 70.6% of moderate to severe patients, which was an independent predictor of severe clinical course in logistic regression analysis (48). In addition, vitamin D deficiency was found to increase the severity of acute respiratory distress syndrome in COVID-19 and suggested that vitamin D supplementation could inhibit hyperinflammation by affecting the inflammatory response of macrophages and myeloid-derived suppressor cells reactions, playing a role in the treatment of COVID-19 (49). Growing evidence supported the potential value of 25(OH)D as a biomarker and adjunctive preventive therapeutic measure in the prevention, treatment, and prognosis of COVID-19 (50).

Due to the limited number of journals included in the PubMed database and a lag in searchable information, it is inevitable that some publications were missed in the search, but the impact on the overall structure of the visualization network was relatively small. Vitamin D is a steroid with pleiotropic actions. Further in-depth research will bring more challenges and greater opportunities to researchers in this field.

## Data Availability Statement

The original contributions presented in the study are included in the article/supplementary material, further inquiries can be directed to the corresponding author/s.

## Author Contributions

XL: preparing the table and figures and drafting the manuscript. CWe: drafting the manuscript, reviewing, editing, conceptualization, and revising. FW and CWa: reviewing and editing the manuscript. All authors contributed to the article and approved the submitted version.

## Conflict of Interest

The authors declare that the research was conducted in the absence of any commercial or financial relationships that could be construed as a potential conflict of interest.

## Publisher's Note

All claims expressed in this article are solely those of the authors and do not necessarily represent those of their affiliated organizations, or those of the publisher, the editors and the reviewers. Any product that may be evaluated in this article, or claim that may be made by its manufacturer, is not guaranteed or endorsed by the publisher.
